# Are Direct Anticoagulants Safer in Traumatic Brain Injury Compared to Warfarin?

**DOI:** 10.1007/s12028-019-00912-3

**Published:** 2020-02-24

**Authors:** Katja E. Wartenberg

**Affiliations:** NeuroIntensive Care Unit, Department of Neurology, University of Leipzig, Liebigstrasse 20, 04103 Leipzig, Germany

Increasing application of direct oral anticoagulants (DOACs) requires new insight into management concepts in traumatic brain injury (TBI). Isolated TBI is more frequently seen in the elderly population [1] which is at higher risk for atrial fibrillation and cardioembolic events. Thus, TBI associated with oral anticoagulation will be more prevalent. Shin et al. present a retrospective evaluation of a larger patient population admitted with TBI linked to oral anticoagulation between 2010 and 2017 at their institution [2]. In this analysis, 136 patients on oral anticoagulation , 39 on DOACs, and 97 on vitamin K antagonists (VKA) were identified. The impact of DOACs and warfarin on hematoma expansion, neurological deterioration, and short-term outcome is compared. For the greater part of this time period, reversal strategies for DOACs according to current standards were not available [3].

Limited data are available about management and outcome of TBI in patients on DOACs; all reports except for one were retrospective and included up to 800 patients with up to 183 patients on DOACs indicating decreased rates of in-hospital death and intracranial hemorrhage in patients on DOACs compared to VKA [4–8]. Only one study described less functional dependence at discharge and a reduction of hemorrhage expansion in patients with TBI and previous DOAC use compared to VKA [8].

In this study, DOAC use prior to TBI was linked to more favorable disposition and functional outcome at discharge according to the Glasgow outcome scale compared to patients on VKA prior to TBI [2]. This difference is not explained by a difference in neurological deterioration, in hemorrhage expansion within 48 h after admission or in in-hospital mortality. In most patients on DOACs, anticoagulation was not even reversed. However, subdural hematoma size and intraparenchymal hematoma volumes were significantly larger in patients on VKA compared to DOACs. The VKA patient group also showed a higher prevalence of arterial hypertension and a trend to higher rates of chronic renal failure, both factors that tend to aggravate intracranial hemorrhages.

The study has two major strengths: (1) application of predefined standards for measurement of hemorrhage expansion for subdural, intraparenchymal, and intraventricular hematomas as well as of the modified Fisher scale for subarachnoid hemorrhage on standardized neuroimaging and (2) report and definition of neurological deterioration after TBI.

The main limitations of the study are the small sample size and the lack of long-term follow-up data for functional and other outcomes such as cognitive impairment and quality of life. The retrospective design constrains the analysis to the available data in the registry. Important predictors of outcome after TBI and confounders, for example cognitive impairment prior to TBI, dosage and time of last administration of the anticoagulant, and hypothermia on admission were not part of the multivariate analysis. Therefore, generalizability of the results to all TBI patients on anticoagulation is restricted. Management strategies may also have changed over a time span of 8 years.

While this study represents a well-done contribution to further knowledge in this field, further research is required to learn more about the effect of DOACs on cerebral hemorrhages in TBI and the need for DOAC effect reversal, the optimal time point for application of the reversal agent in relationship to the last DOAC application prior to TBI, given the cost and side effects of the approved DOAC reversal agents. A large prospective registry similar to RETRACE for anticoagulation-related intracerebral hemorrhage [9] may be sufficient to provide some answers.
